# Comparison of Short-Term Outcomes of Tetralogy of Fallot Repair Between Preserved Pulmonary Valve Annulus and Transannular Patch

**DOI:** 10.7759/cureus.106176

**Published:** 2026-03-31

**Authors:** Md Raisul Islam, Mushtaq Mutashid Muhib, Mohammad Sharifuzzaman, Deepta Majumder

**Affiliations:** 1 Cardiac/Thoracic/Vascular Surgery, National Heart Foundation Hospital and Research Institute, Dhaka, BGD; 2 Medicine, East West Medical College, Dhaka, BGD; 3 Cardiac Surgery, National Heart Foundation Hospital and Research Institute, Dhaka, BGD; 4 Cardiology, Bangladesh Medical University, Dhaka, BGD

**Keywords:** polytetrafluoroethylene, ptfe valve, pulmonary valve annulus, right ventricular function, tetralogy of fallot, transannular patch

## Abstract

Background: Tetralogy of Fallot (TOF) repair may involve either preservation of the pulmonary valve annulus (PVA) or use of a transannular patch (TAP) with a polytetrafluoroethylene (PTFE) bicuspid pulmonary valve. This study aimed to compare short-term clinical and echocardiographic outcomes of TOF repair using preserved PVA versus TAP with PTFE valve.

Methods: This prospective, comparative, cross-sectional study was conducted from January 2022 to December 2023 at the National Heart Foundation Hospital & Research Institute, Dhaka. A total of 46 TOF patients were enrolled: 23 underwent PVA-preserving repair (Group A) and 23 received TAP with PTFE bicuspid valve (Group B). Data on postoperative ventilation time, vasoactive inotrope score (VIS), intensive care unit (ICU) stay, morbidity, and echocardiographic parameters were collected up to 12 weeks post discharge. Statistical analysis was performed using Mann-Whitney U and Fisher’s exact tests.

Results: Group A demonstrated significantly shorter ventilation time (median 5.0 vs. 19.0 hours, p = 0.001), lower VIS (median 9.0 vs. 13.5, p = 0.002), and reduced ICU stay (median 6.0 vs. 7.0 days, p = 0.023). The tricuspid regurgitation peak pressure gradient was significantly lower in Group A at all follow-up points (p < 0.05). No significant differences were observed in overall morbidity, mortality, or incidence of pulmonary regurgitation and right ventricular dysfunction between groups, and no patient required immediate redo surgery for residual right ventricular outflow tract obstruction.

Conclusion: PVA-preserving TOF repair is associated with better early recovery metrics. However, TAP with a PTFE bicuspid valve remains a safe and effective alternative when valve preservation is not feasible.

## Introduction

Tetralogy of Fallot (TOF) is the most common cyanotic congenital heart disease (CHD), with a global incidence of approximately 0.34 per 1000 live births and accounting for about 14% of congenital cardiac anomalies in Bangladesh [1]. It comprises four anatomical abnormalities: ventricular septal defect (VSD), pulmonary stenosis, overriding aorta, and right ventricular (RV) hypertrophy. The surgical correction of TOF is well established and primarily aims to relieve right ventricular outflow tract (RVOT) obstruction and close the VSD. However, the approach to relieving RVOT obstruction significantly influences postoperative outcomes, particularly pulmonary valve function and RV remodeling.

One critical determinant in TOF repair is whether the pulmonary valve annulus (PVA) can be preserved or whether a transannular patch (TAP) may be required to relieve RVOT obstruction. TAP is often used in patients with hypoplastic PVA and effectively alleviates obstruction but at the cost of compromising pulmonary valve integrity, leading to varying degrees of pulmonary regurgitation (PR). PR, in turn, may cause progressive RV dilation, low cardiac output, arrhythmias, and eventually right heart failure or sudden cardiac death, particularly in long-term survivors [2]. To mitigate these complications, reconstructive techniques using synthetic materials such as polytetrafluoroethylene (PTFE) have been explored. PTFE bicuspid pulmonary valves have shown promise in reducing PR severity while offering ease of reproducibility and biocompatibility [3].

The literature reveals mixed findings. Some studies report favorable outcomes with PTFE valve reconstructions in terms of valve competence and RV performance over a short-term follow-up [4,5], while others indicate limited durability and moderate PR even with synthetic valve replacement [6]. Notably, Bangladeshi data remain sparse in this domain, particularly involving direct comparisons between preserved PVA and PTFE bicuspid TAP repairs in TOF patients.

Addressing this gap, this study aimed to compare the short-term outcomes of complete TOF repair using PVA preservation versus TAP reconstruction with a PTFE bicuspid pulmonary valve. The primary outcome measures were early postoperative recovery parameters, including duration of mechanical ventilation, vasoactive inotrope requirement, and length of ICU stay. Secondary outcomes included in-hospital morbidity and mortality, and echocardiographic parameters assessed up to 12 weeks after discharge. Findings from this study may guide surgical decision-making, improve postoperative management, and inform long-term follow-up strategies in the Bangladeshi population and comparable settings globally.

Literature review

Historical Context of TOF Repair

Surgical treatment for TOF began with palliative shunt procedures in the 1940s, followed by complete intracardiac repair in the 1950s. The evolution of open-heart surgery significantly improved survival, transforming TOF into a treatable condition with a favorable long-term prognosis [7,8].

Pulmonary Valve Annulus Preservation

Preservation of the PVA aims to maintain native valve function and avoid PR. Techniques such as commissurotomy, delamination, and balloon dilation have been used to expand mildly hypoplastic annuli [9]. Studies have reported better RV function, reduced PR, and shorter ICU stays with PVA [10-12]. However, anatomical suitability limits its applicability.

Transannular Patch With PTFE Bicuspid Valve

The TAP technique remains essential for severe annular hypoplasia. While traditional TAP without valve reconstruction leads to significant PR, the incorporation of PTFE bicuspid valves shows encouraging results. Rawat et al. and Lee et al. demonstrated high freedom from reintervention and structural valve deterioration [3,4]. However, Nunn et al. (2008) cautioned about limited long-term valve competence [6].

Comparative Outcomes

Studies comparing PVA and TAP highlight that PVA yields superior outcomes in terms of PR and RV function, though not always feasible. TAP with PTFE bicuspid valve offers an alternative with potentially lower PR and better hemodynamics than conventional TAP [5,13]. Nevertheless, findings remain inconclusive regarding early postoperative metrics like ventilation duration, inotropic support, and morbidity.

Gaps and Rationale

Available evidence focuses on mid-to-long-term outcomes. There remains a paucity of studies comparing short-term clinical and echocardiographic outcomes following TOF repair using TAP with PTFE bicuspid valve versus PVA preservation, particularly in the Bangladeshi context. This study aims to bridge that gap with institutionally collected prospective data, offering critical insight into early post-surgical prognosis.

## Materials and methods

Study design and settings

This prospective, observational, comparative study was conducted at the department of pediatric cardiac surgery of a tertiary specialized cardiac hospital in Dhaka. The study spanned from January 2024 to December 2024. The design allowed the evaluation of short-term outcomes in two different surgical approaches to TOF repair within a defined follow-up period of 12 weeks after hospital discharge.

Study area and population

Eligible patients were identified consecutively during the study period according to predefined inclusion and exclusion criteria. Allocation to Group A or Group B was not randomized. The operative approach was determined by the attending surgeon on the basis of preoperative echocardiographic findings, intraoperative anatomical assessment, and the feasibility of preserving the PVA. Patients in whom the annulus and RVOT anatomy were considered suitable for valve preservation underwent PVA-preserving repair (Group A), whereas patients with greater annular hypoplasia or anatomy unsuitable for preservation underwent TAP reconstruction with PTFE bicuspid pulmonary valve (Group B).

Sample size and sampling technique

The sample size for this study was determined based on prior evidence from two relevant studies. According to Toure et al. (2022), the proportion of patients in Group A was 52% [14], while Wankhade et al. (2019) reported that the proportion in Group B was 89.2% [15]. Using these proportions (P1 = 0.52 and P2 = 0.892), the sample size calculation incorporated a significance level (α) of 0.05 for a two-tailed test (Zα/2 = 1.96) and a power (1−β) of 80% (Zβ = 0.84). Applying the standard formula for comparing two proportions, the estimated sample size per group was calculated to be approximately 22.3. This figure was rounded up to 23 participants in each group to ensure adequate power assessment. In line with this calculation, a total of 46 patients who underwent complete repair of TOF were considered for enrollment in this study. Therefore, the study should be interpreted as an observational comparative study with limited power for some individual outcome measures.

Inclusion and exclusion criteria

Group A included patients aged 1-26 years of either sex who underwent TOF repair without a TAP, including pulmonary valve-sparing procedures or separate RVOT and main pulmonary artery (MPA) patch reconstructions. Group B included patients aged 1-25 years who underwent TOF repair using a TAP with a PTFE bicuspid pulmonary valve. All participants were enrolled with ethical approval and informed consent. Exclusion criteria for both groups included TOF with pulmonary atresia, non-confluent pulmonary arteries, atrioventricular septal defect, absent pulmonary valve syndrome, previous palliative operations (e.g., Blalock-Taussig shunt), emergency TOF repair, TOF with double outlet right ventricle (DORV), and repairs involving TAP with a unicuspid valve. Additionally, Group A excluded patients who received TAP or PTFE valve reconstruction, while Group B excluded those who had valve-sparing repairs or RVOT-MPA patch without TAP.

Data collection tools

Data were collected prospectively by the principal researcher using a pretested semi-structured English questionnaire, a structured checklist, and a review of hospital records. Sources included patient files, operative notes, ICU charts, echocardiography reports, and follow-up records. Where necessary, additional information was obtained through face-to-face interviews with parents or guardians. Baseline demographic, clinical, echocardiographic, intraoperative, and postoperative data were recorded in a standardized manner.

Study variables

The variables examined in this study included a range of dependent, independent, and potential confounding factors. The dependent variables focused on short-term postoperative outcomes such as duration of mechanical ventilation, length of ICU stay, duration of inotropic support, occurrence of complications, including low cardiac output and arrhythmias, as well as mortality. Echocardiographic outcomes such as pulmonary regurgitation, RV function, and RVOT gradient were assessed at discharge and at four weeks and 12 weeks post discharge. Independent variables encompassed demographic characteristics like age, sex, and body surface area, alongside clinical parameters such as oxygen saturation and hematocrit levels. Additionally, preoperative, intraoperative, and postoperative echocardiographic and surgical attributes such as the McGoon ratio, pulmonary valve z-scores, and cardiopulmonary bypass time were considered. Confounding variables included baseline cardiac anomalies, anatomical variations, intraoperative findings, and pre-existing physiological conditions that might influence surgical outcomes.

Surgical technique

**Figure 1:**
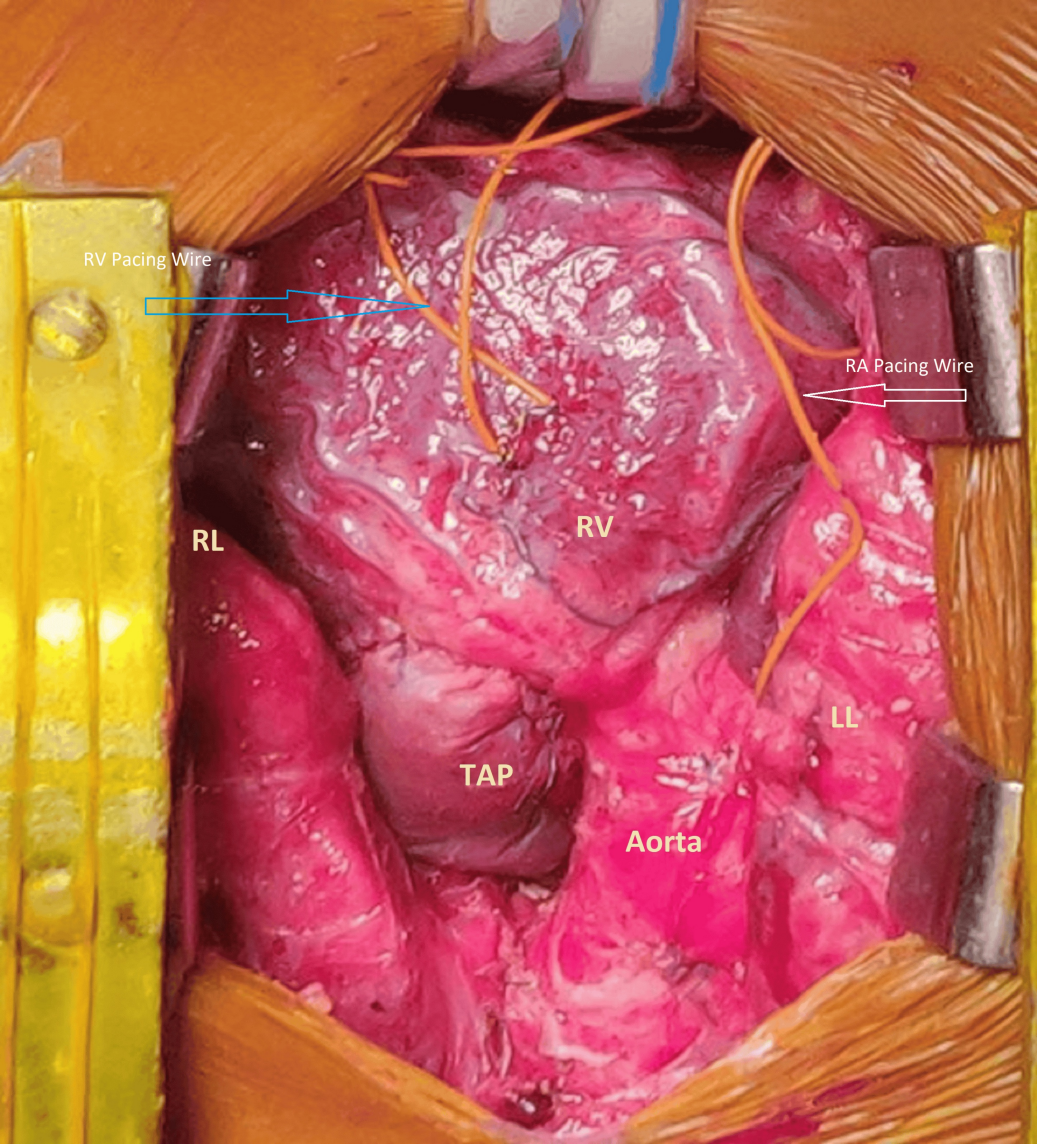
Intraoperative view following complete repair of tetralogy of Fallot. This picture is in an upward cephalic and a downward caudal position. TAP: transannular patch; RA: right atrium; RV: right ventricle; RL: right lung; LL: left lung.

**Figure 2:**
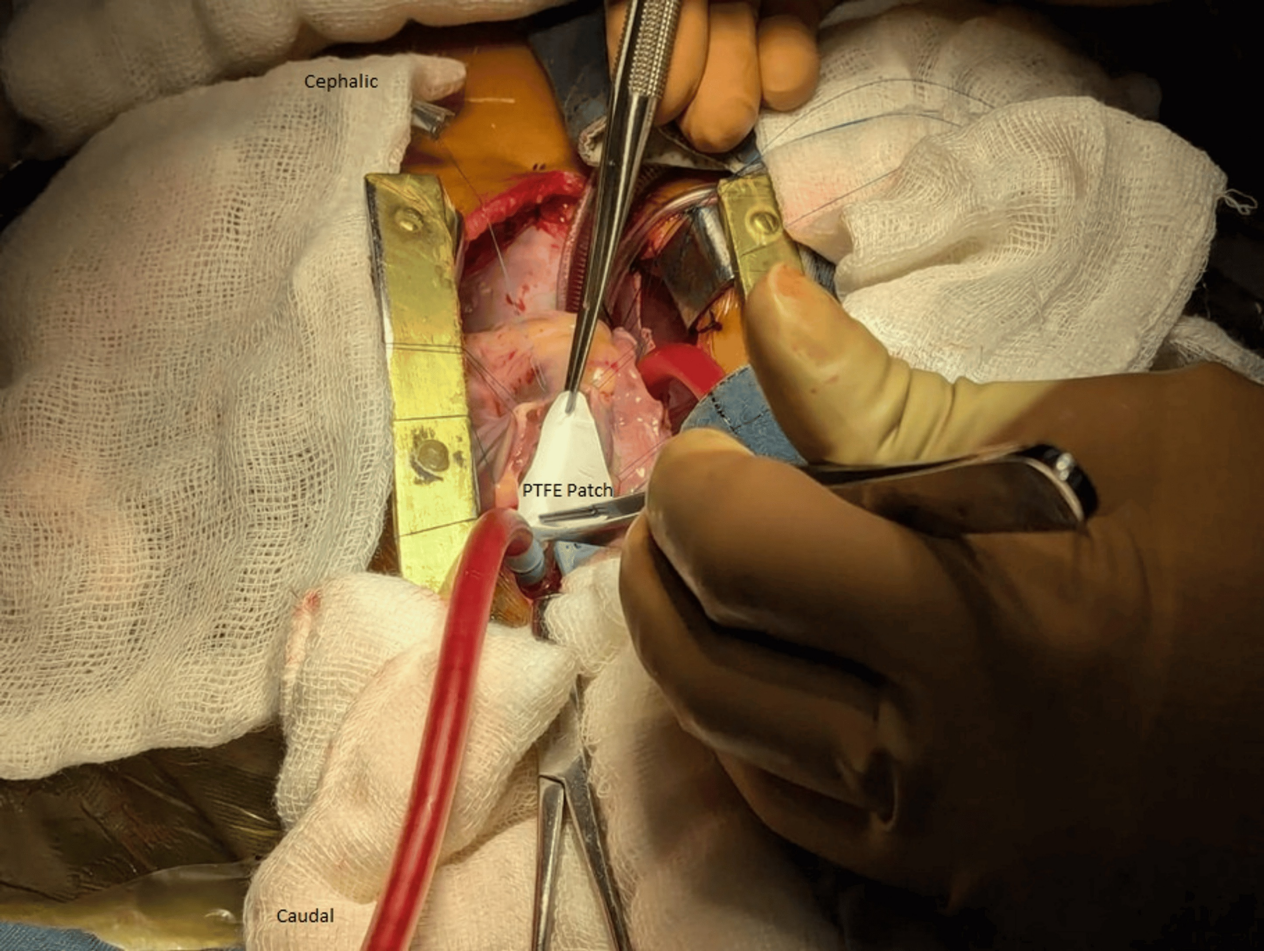
Intraoperative placement of a PTFE patch. PTFE: a patch made of polytetrafluoroethylene.

All patients underwent surgery through a standard median sternotomy under moderate hypothermia (28-32°C). Autologous pericardium was harvested and treated with 0.625% glutaraldehyde for use in patching. Following aortic and bicaval cannulation, cardiopulmonary bypass was initiated, and the heart was arrested using antegrade cold blood cardioplegia. The right atrium was opened for ventricular septal defect closure using a Dacron patch. In Group A, the pulmonary valve was preserved, or commissurotomy was performed if fused. In Group B, when the valve was hypoplastic or dysplastic and annular preservation was not feasible, pulmonary valvotomy was followed by reconstruction using a hand-sewn PTFE bicuspid pulmonary valve according to the Graham-Nunn technique, in which a PTFE valved mechanism is fashioned for the RVOT to improve pulmonary competence after TAP enlargement [6]. The PTFE valve was fashioned intraoperatively on the operating table by the operating surgeon according to annular size and RVOT anatomy. The reconstructed valve was then incorporated into the TAP repair to provide a degree of pulmonary valve competence. Because this was a hand-fashioned reconstruction rather than a standardized commercial prosthesis, some technical variation related to surgeon judgement and intraoperative anatomy was unavoidable (Figures 1, 2).

Data collection and quality control

Data were collected prospectively by the researcher himself. Standardized protocols were used to maintain uniformity during the entire study. Postoperative data were collected during hospitalization and at scheduled follow-up visits (four and 12 weeks). Quality control included data cleaning, categorization, and coding. Consistency was ensured through cross-verification of hospital records and direct validation with clinical data to eliminate errors or missing information.

Data analysis

All statistical analyses were performed using SPSS version 26 (IBM Corp., Armonk, NY). Descriptive statistics were used to summarize baseline, intraoperative, and postoperative variables. Categorical variables were presented as frequencies and percentages. Continuous variables were examined for distribution and, where not normally distributed, were summarized as medians with interquartile ranges. Because several continuous study variables, including ventilation time, vasoactive inotrope score, and ICU stay, showed non-normal distribution and the sample size was relatively small, between-group comparisons for continuous variables were performed using the Mann-Whitney U test. Categorical variables were compared using the chi-square test when assumptions for expected cell counts were met and Fisher’s exact test when cell counts were small. A p-value of ≤0.05 was considered statistically significant.

Outcome measures

The primary endpoint domain of the study was early postoperative recovery, assessed by duration of mechanical ventilation, vasoactive inotrope score, and length of ICU stay. Secondary outcomes included in-hospital morbidity and mortality, as well as echocardiographic outcomes assessed at discharge and at four and 12 weeks after discharge, including pulmonary regurgitation, pulmonary stenosis, right ventricular dysfunction, RVOT-pulmonary artery (PA) gradient, and tricuspid regurgitation (TR) peak pressure gradient (PPG).

## Results

The baseline characteristics of the study population showed no statistically significant differences between Group A and Group B across age, gender, or body surface area (BSA). Most patients in both groups were aged between one and five years, with a few older participants. Male patients slightly predominated in both groups. The majority of respondents in both groups had BSA values in the 0.34-0.74 m² range. Fisher’s exact test and chi-square test results confirmed that none of these baseline differences were statistically significant (p > 0.05), indicating that the groups were demographically and anthropometrically comparable (Table 1).

**Table 1:** Baseline comparison of age, gender, and body surface area between groups A and B. * P-value was determined by Fisher’s exact test; ** p-value was determined by chi-square test. P-value > 0.05 is not statistically significant. Degrees of freedom (df) refer to the number of independent values or quantities that can be assigned to a statistical distribution.

Variable	Category	Group A (n = 23)	Group B (n = 23)	χ^2^	Degrees of freedom (df)	p-value
Age (years)	1-5	11 (47.8%)	14 (60.9%)	1.006	2	0.649*
	6-20	10 (43.5%)	8 (34.8%)			
	21-25	2 (8.7%)	1 (4.3%)			
Gender	Male	13 (56.5%)	14 (60.9%)	0.09	1	0.765^**^
	Female	10 (43.5%)	9 (39.1%)			
Body surface area (m²)	0.34-0.74	14 (60.9%)	18 (78.3%)	1.658	2	0.476*
	0.75-1.14	5 (21.7%)	3 (13.0%)			
	1.15-1.56	4 (17.4%)	2 (8.7%)			

In Table 2, the comparison of preoperative variables between Group A and Group B showed no statistically significant differences across all measured parameters. Oxygen saturation (SpO₂) distribution was comparable in both groups, with the majority falling in the 65-78% range. Hematocrit levels were also similarly distributed, most frequently within the 34-40% range. Echocardiographic findings, including the PVA Z-score, RVOT-PA gradient, and TR peak pressure gradient, showed no significant variation between groups. Additionally, the median McGoon ratio was identical (1.90) in both groups, further supporting baseline comparability.

**Table 2:** Comparison of preoperative variables between groups A and B. * P-values determined by Fisher’s exact test for categorical variables and ** p-values determined by the Mann-Whitney U test for continuous variables. P-value > 0.05 is not statistically significant IQR: interquartile range (Q1-Q3); df: degree of freedom; S.E.: standard error; SpO_2_: oxygen saturation; PPG: peak pressure gradient is a key echocardiographic measurement used to estimate the pressure difference between the right ventricle and the right atrium; RVOT-PA: right ventricular outflow tract (RVOT) is the infundibular, superior portion of the right ventricle that connects to the pulmonary artery (PA); PV: the pulmonary valve is a semilunar valve located at the junction of the right ventricle and the pulmonary artery (PA). It regulates the flow of blood from the right ventricle into the pulmonary circulation during systole.

Variables	Group A (n = 23)	Group B (n = 23)	U/χ^2^	df/S.E.	p-value
SpO₂ (%)			0.377	2	1.00*
50-64	1 (4.3%)	1 (4.3%)			
65-78	15 (65.2%)	15 (60.9%)			
79-93	7 (30.4%)	8 (34.8%)			
Hematocrit (%)			0.358	2	0.866*
34-40	9 (39.1%)	11 (47.8%)			
41-46	6 (26.1%)	5 (21.7%)			
47-52	8 (34.8%)	7 (30.4%)			
Z-score for PV annulus [16]	-3.20 (-5.0 to -2.0)	-5.0 (-7.0 to -2.3)	193.5	45.3	0.117**
RVOT-PA gradient (mmHg) [17]	70.0 (65.0-88.0)	74.0 (66.0-80.0)	271	45.3	0.886**
TR (PPG) (mmHg) [18]	57.0 (50.0-63.0)	55.0 (50.0-63.0)	249.5	45.4	0.741**
McGoon ratio [19]	1.90 (1.60-2.40)	1.90 (1.60-2.19)	247.5	45.3	0.708**

The median cardiopulmonary bypass (CPB) time was 93.0 minutes in Group A and 108.0 minutes in Group B (Table 3). Similarly, the median aortic cross-clamp time was 70.0 minutes for Group A and 82.0 minutes for Group B. Statistical analysis using the Mann-Whitney U test indicated no significant differences between the groups for either CPB or aortic cross-clamp times (p > 0.05).

**Table 3:** Comparison of peroperative variables between group A and B. P-values were calculated using the Mann-Whitney U test. P-value > 0.05 is not statistically significant. IQR: interquartile range (Q1-Q3); S.E.: standard error.

Peroperative variables	Group A (n = 23), Median (IQR)	Group B (n = 23), Median (IQR)	U	S.E.	p-value
Cardiopulmonary bypass (CPB) time (minutes)	93.0 (84.0-123.0)	108.0 (94.0-139.0)	331.5	45.5	0.141
Aortic cross-clamp time (minutes)	70.0 (57.0-88.0)	82.0 (61.0-103.0)	312	45.5	0.297

The median ventilation time was notably shorter in Group A at 5.0 hours compared to 19.0 hours in Group B (p = 0.001). Similarly, the median vasoactive inotrope score was lower in Group A at 9.0 versus 13.5 in Group B (p = 0.002). Additionally, the median ICU stay was shorter in Group A, with 6.0 days compared to 7.0 days in Group B (p = 0.023). These statistically significant results indicate that patients in Group A experienced better early postoperative recovery than those in Group B (Table 4).

**Table 4:** Comparison of postoperative variables between groups A and B. * Significant at p < 0.05 (Mann-Whitney U test). IQR: interquartile range (Q1-Q3); S.E.: standard error; ICU: intensive care unit.

Variable	Group A (n = 23), Median (IQR)	Group B (n = 23), Median (IQR)	U	S.E.	p-value
Ventilation time (hours)	5.0 (3.0-17.0)	19.0 (13.0-65.0)	422	45.4	0.001*
Vasoactive inotrope score [20]	9.0 (8.0-13.0)	13.5 (10.0-23.0)	405.5	45.2	0.002*
ICU stay (days)	6.0 (4.0-7.0)	7.0 (6.0-10.0)	366	44.7	0.023*

In Table 5, the comparison of in-hospital morbidity and mortality between the two groups showed no statistically significant differences in complications (all p-values > 0.05). The median hematocrit levels at discharge, four weeks, and 12 weeks post discharge were similar between groups A and B, with no significant differences observed (p = 0.45, 0.66, and 0.55, respectively). Likewise, the RVOT-PA gradient measured at the same time points showed no significant difference between the groups (p = 0.589, 0.613, and 0.732). However, the TR (PPG) values were significantly lower in group A compared to group B at discharge (p = 0.017), at four weeks (p = 0.025), and at 12 weeks (p = 0.011), indicating a statistically significant difference in this parameter. Pulmonary regurgitation distribution between the groups did not differ significantly at discharge, four weeks, or 12 weeks’ follow-up (p = 0.052, 0.058, and 0.058). Pulmonary stenosis incidence was also comparable, with no significant differences across all time points (p = 1.0, 0.429, and 0.599). Finally, right ventricular dysfunction was rare and showed no statistically significant difference between groups at discharge, four weeks, or 12 weeks (p = 1.0, 1.0, and 0.49), and free pulmonary regurgitation was included as a separate category within postoperative pulmonary regurgitation grading. Overall, except for TR (PPG), no other measured postoperative parameters or complications demonstrated statistically significant differences between the two groups.

**Table 5:** Comparison of postoperative outcomes and echocardiographic parameters between Group A (valve-preserving repair) and Group B (transannular patch with PTFE bicuspid pulmonary valve). * Significant at p < 0.05. U: Mann-Whitney U test; E: Fisher's exact test; PTFE: polytetrafluoroethylene; IQR: interquartile range; S.E.: standard error; RVOT: right ventricular outflow tract; PA: pulmonary artery; PPG: peak pressure gradient; TR: tricuspid regurgitation.

Parameter	Group A (n = 23)		Group B (n = 23)	χ^2^/U	df/S.E.	p-value
In-hospital morbidity & mortality						
Low cardiac output syndrome	0 (0.0%)		1 (4.3%)	1.022	1	1.0^E^
Re-intubation	1 (4.3%)		2 (8.7%)	0.357	1	1.0^ E^
Respiratory complications	2 (8.7%)		5 (21.7%)	1.516	1	0.414^ E^
Pneumothorax	1 (4.3%)		0 (0.0%)	1.012	1	1.0^ E^
Arrhythmia	2 (8.7%)		3 (13.0%)	0.224	1	1.0^ E^
Acute renal failure	0 (0.0%)		1 (4.3%)	1.022	1	1.0 ^E^
Postoperative dialysis	0 (0.0%)		1 (4.3%)	1.022	1	1.0 ^E^
Neurological complications	1 (4.3%)		0 (0.0%)	1.022	1	1.0^ E^
Deep wound infection	1 (4.3%)		1 (4.3%)	0	1	1.0^ E^
Operative mortality	1 (4.3%)		1 (4.3%)	0	1	1.0^ E^
Hematocrit, Median (IQR)						
At discharge	39.68 (37.20-40.0)		39.06 (34.72-40.92)	239.5	44.9	0.45^U^
At 4 weeks	38.30 (38.75-39.95)		38.30 (38.13-40.0)	293.5	44.9	0.66^ U^
At 12 weeks	38.0 (37.0-39.50)		38.0 (36.2-39.50)	291.5	44.7	0.55^ U^
RVOT-PA gradient (mmHg), Median (IQR)						
At discharge	16.14 (10.75-20.0)		17.5 (9.0-24.5)	283	45.4	0.59^ U^
At 4 weeks	12.0 (9.5-18.5)		15.5 (7.75-24.0)	286.5	45.2	0.61^ U^
At 12 weeks	14.0 (9.5-16.5)		13.5 (7.75-23.0)	277.5	45.2	0.73^ U^
TR (PPG) (mmHg), Median (IQR)						
At discharge	16.0 (13.0-24.0)		21.0 (16.0-35.0)	366	45.3	0.017*^ U^
At 4 weeks	14.0 (12.0-20.0)		19.0 (14.0-30.0)	358	45.2	0.025*^ U^
At 12 weeks	13.0 (12.0-18.0)		17.0 (14.0-30.0)	372	45.2	0.011*^ U^
Pulmonary regurgitation						
No regurgitation	7 (31.8%)		1 (4.5%)	9.686	4	0.052^ E^
Trivial regurgitation	4 (18.2%)		1 (4.5%)			
Mild regurgitation	11 (50.0%)		17 (77.3%)			
Moderate regurgitation	0 (0.0%)		2 (9.1%)			
Severe regurgitation	0 (0.0%)		0 (0.0%)			
Free-flow regurgitation	0 (0.0%)		1 (4.5%)			
Pulmonary stenosis						
No stenosis	20 (90.9%)		20 (90.9%)	0.978	2	1.0^ E^
Trivial stenosis	0 (0.0%)		1 (4.5%)			
Mild stenosis	2 (9.1%)		2 (9.1%)			
Right ventricular dysfunction						
At discharge	0 (0.0%)		1 (4.5%)	1.023	1	1.0^ E^
At 4 weeks	0 (0.0%)		1 (4.5%)	1.023	1	1.0^ E^
At 12 weeks	0 (0.0%)		2 (9.1%)	2.095	1	0.49^ E^

## Discussion

The surgical correction of TOF has evolved significantly over recent decades, with growing emphasis on long-term cardiac function and quality of life. This study addresses the crucial clinical question of whether preserving the PVA or using a TAP with a PTFE bicuspid pulmonary valve provides more favorable short-term outcomes. The findings reinforce the relevance of tailoring the surgical approach based on anatomical feasibility and anticipated postoperative sequelae. The findings suggest that both techniques can be performed with acceptable short-term safety; however, interpretation of comparative effectiveness requires caution because operative allocation was surgeon-directed and anatomically influenced rather than randomized.

In clinical practice, the use of TAP often becomes necessary in patients with severely hypoplastic pulmonary valve annuli. While effective in relieving RVOT obstruction, this approach is typically associated with varying degrees of PR, which can progress to RV dilation and dysfunction. In response to this challenge, valve reconstruction techniques using PTFE have been developed to reduce PR while maintaining RV function. Studies such as those by Lee et al. and Rawat et al. have shown that PTFE bicuspid valves provide acceptable short-term performance. Rawat et al. reported that bicuspid PTFE membrane valves reduced immediate postoperative central venous pressure, facilitated earlier extubation, improved pulmonary competence, and did not increase the RVOT gradient during short-term follow-up when compared with transannular pericardial patch reconstruction [3,4]. Our findings add short-term observational data from a Bangladeshi cohort up to 12 weeks after surgery.

Preservation of the PVA, when technically feasible, has gained favor due to its potential to reduce the incidence of PR and its subsequent effects on RV remodeling. Previous studies emphasize the long-term benefits of PVA in minimizing right heart dilatation and maintaining better hemodynamics [10,21]. In our findings, while echocardiographic gradients (such as TR PPG) showed statistically significant differences favoring annulus preservation, the actual clinical impact on morbidity and mortality remained comparable between groups over the short-term period. This suggests that although annulus preservation may be associated with more favorable short-term hemodynamic findings in anatomically suitable cases, TAP with PTFE reconstruction remains a viable alternative with acceptable early outcomes when annular preservation is not feasible.

Importantly, the incorporation of PTFE bicuspid valves in TAP repair seeks to mitigate the traditionally poor hemodynamic profile of TAP alone. Previous evidence suggests that valved reconstruction added to a TAP can reduce immediate and short-term PR compared with a standard non-valved TAP. However, the present study did not compare different leaflet configurations, such as monocusp, bicuspid, or trileaflet reconstruction; therefore, no conclusion can be drawn from our data regarding the superiority of one valved design over another. The literature supports the mechanical stability of such valves, with low rates of structural valve deterioration within the first five years [4,22]. In our study, despite a slightly increased rate of PR in the TAP group, the functional class and absence of significant RV dysfunction in most patients suggest clinical compensation in the early recovery phase. Although overall PR distribution did not reach conventional statistical significance, this finding should be interpreted cautiously. Moderate and free-flow PR occurred only in the TAP with PTFE group, suggesting a directional difference that may be clinically relevant. Given the small sample size, the study was likely underpowered to detect differences in this endpoint, and the absence of statistical significance should not be interpreted as equivalence between the two groups. This observation is consistent with other short-term outcome studies [5,22,23], which highlight that mild to moderate PR can be tolerated without compromising early postoperative recovery.

The TAP with PTFE group showed less favorable early recovery metrics, including longer ventilation time, higher vasoactive inotrope score, and longer ICU stay. This pattern is consistent with the likelihood that patients in the TAP group had greater underlying anatomical complexity at baseline. However, these differences should be interpreted cautiously, as the TAP group likely included patients with more complex or less favorable RVOT and annular anatomy. Therefore, the observed differences may reflect, at least in part, underlying anatomical severity and surgeon-directed case selection rather than the effect of the reconstructive technique alone. These findings align with some studies where authors had pointed out that the perioperative stress response is often greater in TAP cases due to more extensive myocardial and pulmonary artery manipulation, though outcomes normalize in follow-up assessments [15,24]. In resource-constrained settings, PTFE valved reconstructions may also be fashioned intraoperatively on the operating table according to the surgeon’s expertise, which increases the practical applicability of this approach in developing countries [25].

Limitation

This study has several limitations. First, allocation to the two operative groups was not randomized but surgeon-directed according to anatomical feasibility and intraoperative findings, introducing the possibility of selection bias. Patients undergoing PVA-preserving repair were likely to have more favorable baseline annular and RVOT anatomy than those requiring TAP reconstruction. Second, the PTFE bicuspid valve reconstruction followed the Graham-Nunn technique but was fashioned intraoperatively according to the surgeon's judgment and patient anatomy rather than by a fully standardized prefabricated protocol. In a resource-variable setting, this may introduce technical heterogeneity that could influence postoperative performance and limit exact procedural reproducibility across centers. Third, the study was conducted at a single center with a relatively small sample size and short follow-up duration, which limits generalizability. Fourth, the pulmonary vein index (PVI) was not assessed in the present study. Previous studies have reported PVI as a useful early prognostic indicator after TOF repair and as a marker associated with early postoperative recovery, including the risk of adverse early outcomes. Therefore, the absence of PVI assessment limited our ability to evaluate its potential contribution to short-term postoperative prognosis in this cohort.

## Conclusions

PVA-preserving TOF repair was associated with better early recovery measures in this cohort. When annular preservation was not anatomically feasible, TAP with PTFE bicuspid pulmonary valve reconstruction remained a feasible short-term option. However, these findings should be interpreted cautiously because group allocation was non-randomized and anatomically guided, and the pulmonary regurgitation profile appeared less favorable in the TAP group. Larger multicenter studies with longer follow-up are needed to better define the comparative effectiveness and durability of these strategies.
